# Stereo-EEG-guided network modulation for psychiatric disorders: Interactive holographic planning^☆^

**DOI:** 10.1016/j.brs.2023.11.003

**Published:** 2023-12-20

**Authors:** Angela M. Noecker, Jeffrey Mlakar, Kelly R. Bijanki, Mark A. Griswold, Nader Pouratian, Sameer A. Sheth, Cameron C. McIntyre

**Affiliations:** a Department of Biomedical Engineering, Case Western Reserve University, Cleveland, OH, USA; b Department of Biomedical Engineering, Duke University, Durham, NC, USA; c Interactive Commons, Case Western Reserve University, Cleveland, OH, USA; d Department of Neurosurgery, Baylor College of Medicine, Houston, TX, USA; e Department of Neurosurgery, University of Texas Southwestern, Dallas, TX, USA; f Department of Neurosurgery, Duke University, Durham, NC, USA; g Department of Radiology, Case Western Reserve University, Cleveland, OH, USA

**Keywords:** sEEG, DBS, Depression, HoloLens, Visualization

## Abstract

**Background:**

Connectomic modeling studies are expanding understanding of the brain networks that are modulated by deep brain stimulation (DBS) therapies. However, explicit integration of these modeling results into prospective neurosurgical planning is only beginning to evolve. One challenge of employing connectomic models in patient-specific surgical planning is the inherent 3D nature of the results, which can make clinically useful data integration and visualization difficult.

**Methods:**

We developed a holographic stereotactic neurosurgery research tool (HoloSNS) that integrates patient-specific brain models into a group-based visualization environment for interactive surgical planning using connectomic hypotheses. HoloSNS currently runs on the HoloLens 2 platform and it enables remote networking between headsets. This allowed us to perform surgical planning group meetings with study co-investigators distributed across the country.

**Results:**

We used HoloSNS to plan stereo-EEG and DBS electrode placements for each patient participating in a clinical trial (NCT03437928) that is targeting both the subcallosal cingulate and ventral capsule for the treatment of depression. Each patient model consisted of multiple components of scientific data and anatomical reconstructions of the head and brain (both patient-specific and atlas-based), which far exceed the data integration capabilities of traditional neurosurgical planning workstations. This allowed us to prospectively discuss and evaluate the positioning of the electrodes based on novel connectomic hypotheses.

**Conclusions:**

The 3D nature of the surgical procedure, brain imaging data, and connectomic modeling results all highlighted the utility of employing holographic visualization to support the design of unique clinical experiments to explore brain network modulation with DBS.

## 1. Introduction

Deep brain stimulation (DBS) is an investigational therapy for treatment resistant depression (TRD) [1]. Therapeutic response to DBS for TRD has been inconsistent [2], with many examples of excellent outcomes [3,4], but also two industry-sponsored trials that were discontinued due to concerns for lack of efficacy [5,6]. However, recent advances in image-based surgical targeting strategies [7,8] suggest that TRD DBS outcomes can be improved if stimulation is focused on specific axonal pathways in depression-relevant networks [9]. As such, an evolving sub-field of DBS research, known as connectomic DBS modeling, is using connectivity models to help identify patient-specific targets for stimulation [10].

Connectomic modeling studies have helped to expand understanding of the networks that are engaged by DBS [11]. However, human intracranial measurements of depression-relevant networks in TRD subjects are typically limited to a single site of stimulation (i.e. subcallosal cingulate (SCC) or ventral capsule (VC)), without recordings of DBS-evoked responses in other nodes of the network. This clinical limitation hinders opportunities to interrogate the network and dissect the key structures for therapeutic neuromodulation [12]. In response to this limitation, parallel work by Scangos et al. [13] and Sheth et al. [14] have established new surgical paradigms aimed at analyzing the neurocircuitry of TRD with the temporary implantation of stereo-electroencephalography (sEEG) electrodes. The sEEG electrodes are placed in key prefrontal grey matter areas of depression-relevant networks to acquire uniquely valuable electrophysiology data with exquisite spatial precision. In turn, the concepts of sEEG-informed DBS therapy development are providing new opportunities to define symptom-related brain states and stimulation dose-response relationships at the brain network level [15,16].

The ongoing work of clinical trial NCT03437928 (ClinicalTrials.gov) [14], is applying the novel approach of targeting both SCC and VC in TRD patients with permanent DBS electrodes, while also using 10–12 temporary sEEG electrodes, for 10 days of local field potential (LFP) recording after the surgery. These unique experiments enable detailed electrophysiological investigation on the modulation of depression-relevant networks with DBS, and documentation of the spatial distribution of DBS-evoked network activity throughout the prefrontal cortices with sEEG recordings [16]. However, strategic positioning of the DBS electrodes, as well as the sEEG electrodes, in each research subject is essential to maximize the value of the data collection and exploration of the neurocircuitry. Therefore, we identified a need for novel surgical planning and analysis tools that could integrate the 3D anatomy of the patient brain with the latest scientific imaging results on the brain networks of interest. Our response to this technical need was the development of a holographic stereotactic neurosurgery research tool (HoloSNS) that could aid in the planning of complex cases [17]. This report describes our first experiences with the prospective use of HoloSNS in support of clinical trial NCT03437928.

## 2. Methods

### 2.1. HoloSNS

**Fig. 1.**
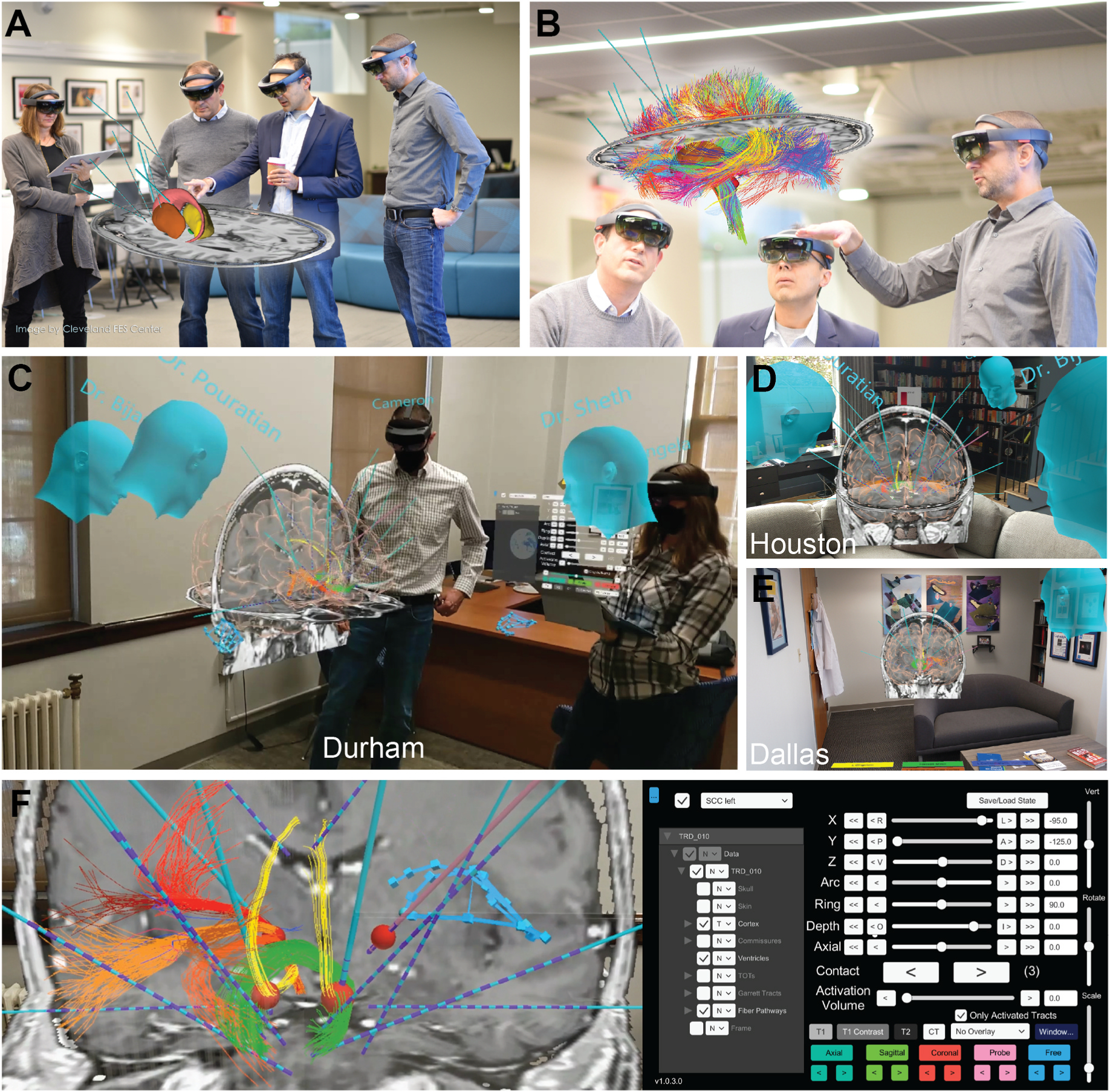
HoloSNS. A,B) Example scenes from the first group-based surgical planning session for the first subject in NCT03437928. C) Example scene from a planning session for the fourth subject implanted in NCT03437928 from McIntyre’s office in Durham, highlighting the transition to remote group-based planning sessions. D) View from Dr. Sheth’s HMD in Houston. E) View from Dr. Pouratian’s HMD in Dallas. F) Interactive selection and positioning of a DBS lead (pink shaft) by an avatar hand (blue wire-frame hand) of a remote participant (Dr. Sheth) within the patient-specific brain model. Red volumes on the DBS leads simulate an activation volume and streamlines that pass through that volume are displayed. An example of the HoloSNS control panel is shown on the right. The hierarchical menu on the left is used to select the model elements for viewing. The upper left drop down menu is used to select the lead for interaction. The stereotactic coordinates for that lead tip and trajectory can then be adjusted via click buttons, sliders, or direct entry. The electrode contact (number in parentheses) for activation volume display can be selected and the size of the activation volume can be adjusted. The lower collection of buttons control the viewing of the MRI/CT datasets.

HoloSNS is currently designed to run on the Microsoft HoloLens 2 (HL2) platform. HL2 is an untethered head-mounted display (HMD) that uses transparent screens and stereoscopic rendering to generate a visual scene that the user sees as a 3D hologram (Fig. 1). The HoloSNS application was developed using the Unity Game Engine and the Mixed Reality Toolkit from Microsoft. The patient-specific modeling infrastructure of HoloSNS is based on the academic software tool StimVision [18], and adapted for use within an augmented reality HMD [17]. Noecker et al. [17] provides a technical description of HoloSNS, which is briefly summarized below.

HoloSNS integrates patient-specific brain imaging datasets and atlases into a group-based holographic visualization environment for interactive neurosurgical planning using connectomic hypotheses (Supplemental Videos 1, 2, 3). The group of users can be local (i.e. in the same room (Fig. 1A)) and/or remote (i.e. in different cities (Fig. 1C, D, E)), but each user can simultaneously interact with the holographic patient model, as well as see and talk with the other users in real time (Supplemental Videos 1, 2, 3). Each HoloSNS user sees the same hologram through their HMD. The hologram is constantly updated as the various users adjust the simulation, including the selection of the imaging data, lead positions, and stimulation modeling results. When multiple users are in the same room, they see each other through the transparent visor of their HMD. When a user is remote, an avatar head identifies their position in the holographic scene for the other users to see (Fig. 1C, D, E). Audio information from remote users is transmitted to the other HMDs via Voice over Internet Protocol (VoIP). The HL2 headsets have built-in speakers that provide 3D audio, so users hear the voices of the remote participants in a manner that is consistent with their position relative to the simulated hologram.

Sensors in the HL2 HMD recognize the hands of the user and avatar representations of their hands are incorporated into the HoloSNS simulation (Fig. 1F). Users can then pinch their index finger and thumb of their avatar hand together around an object (i.e. a MRI slice or an electrode lead) to grab it and interactively move it within the holographic scene. A holographic control panel is used to adjust visualization of the model components in the scene (e.g. turn components on/off). The control panel also runs on a tablet PC that is linked to the HMDs and some users prefer this mode of interaction with the simulation.

### 2.2. Patient-specific HoloSNS models

**Fig. 2.**
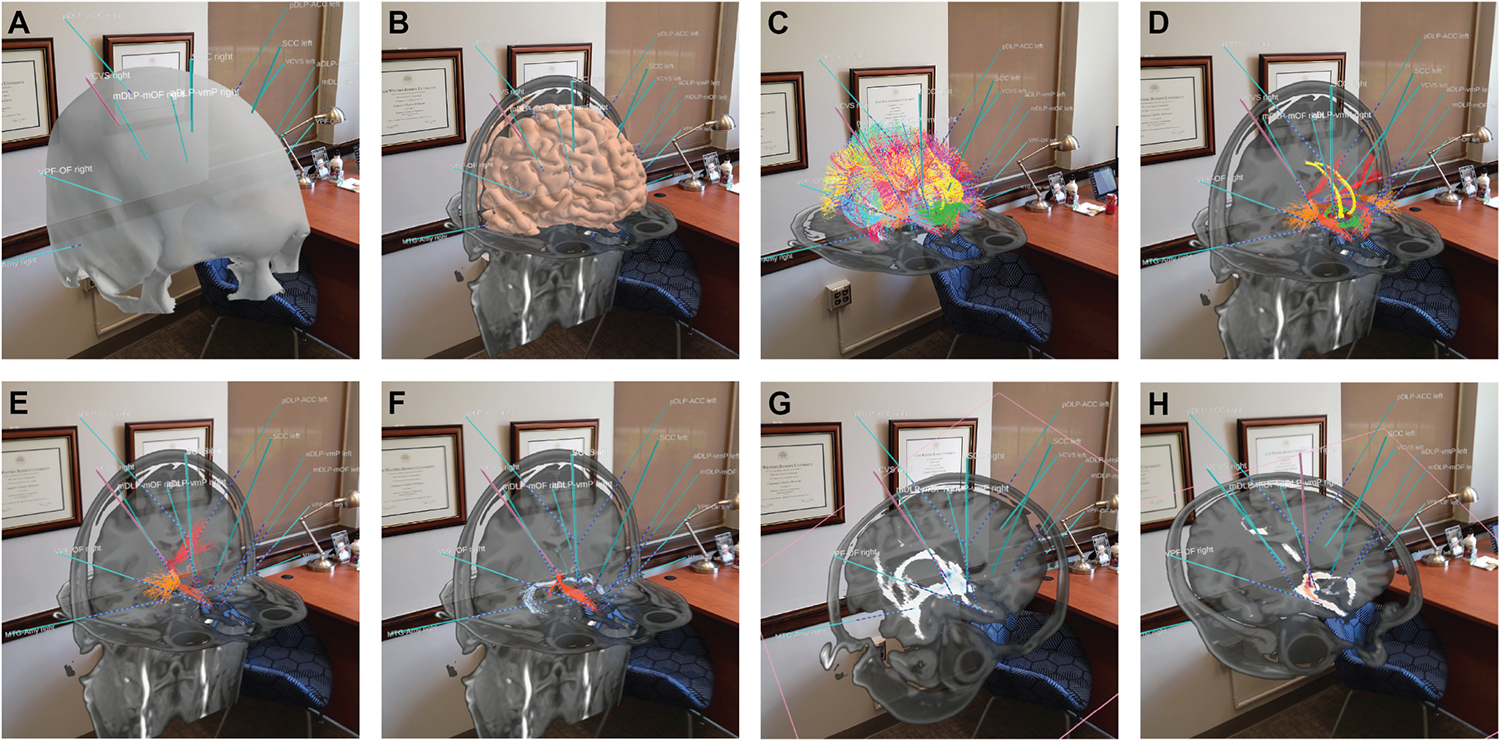
HoloSNS example data visualization. A) Reconstruction of the patient skull with 14 lead trajectories. B) Reconstruction of the pial surface with 14 leads, as well as coronal and axial MRI slices. C) Whole brain anatomically annotated axonal pathway models with 14 leads and an axial MRI slice. D) Simulated pathway activation from all 4 DBS leads. Red/orange pathways project to/from the VC activation volumes to prefrontal cortex. Yellow/green pathways project to/from the SCC activation volumes. E,F) Isolated pathway activation from the right VC DBS lead (pink trajectory) at contact 8 (E), or contact 1(F). G,H) Probabilistic tractography heatmaps overlaid on the MRI slice. These examples display “probes eye view” slices (i.e. perpendicular to the selected lead trajectory) at the level of the 5,6,7 ring of DBS contacts. G) Blue-to-white tractography heatmap for the VC DBS target region. H) Red-to-white tractography heatmap for the SCC DBS target region.

The technical details of the medical imaging datasets acquired for the subjects in clinical trial NCT03437928 were described in Adkinson et al. [16]. For each subject, the imaging data were acquired ~1 month prior to their surgery date. The imaging team members for the study, located at Baylor (Sheth and Bijanki Labs), UTSW (Pouratian Lab), and Duke (McIntyre Lab), then worked together to perform a pre-defined list of study-specific processing steps on the imaging data. Those analyses generated the various datasets that were loaded into HoloSNS for each patient model (Fig. 2). The datasets include probabilistic tractography heat maps from the SCC and VC target regions [8], tractography optimized target volumes [14], anatomical pathway models [19,20], resting state functional MRI areas of correlation/anti-correlation [21], anatomical nuclei volumes [22], as well as pial surface, blood vessel, skull, and scalp reconstructions. These image processing steps were typically completed ~3 weeks prior to the surgery date. Several of these datasets of interest are relatively new research-based items that are not available in traditional surgical planning workstations. Therefore, an important motivation for our use of HoloSNS was the opportunity to directly integrate these study-specific custom-made models with the typical imaging data used in traditional DBS surgical planning.

The anatomical nuclei models and streamline axonal pathway models we included in each patient-specific brain model were derived from atlas-based datasets [19,20,22], which were defined within the coordinate system of the CIT168 brain atlas [22]. Therefore, we co-registered the CIT168 brain volume to the patient brain using an affine transformation matrix and warp field that was created via Advanced Normalization Tools (ANTs) using symmetric normalization (SyN) (http://picsl.upenn.edu/software/ants/). The resulting transform and warp field were applied to the polygonal data of the 3D anatomical nuclei and axonal pathway streamlines to place them into patient-specific space.

All of the image-based modeling results were co-registered to, and visualized within, the original T1w and T2w MRI scans of the patient. The datasets were also processed by the augmented reality team at CWRU to optimize their rendering within the HL2 platform. The patient-specific model was then compiled for the HoloSNS app and loaded onto the HMDs. Finally, the prepared HMDs were shipped out to the team of investigators ~2 weeks prior to the surgery date for each subject.

For each subject, the electrode placement planning team scheduled 2–3 dates/times for the group-based HoloSNS sessions (Fig. 1). We typically spent the first ~2 h planning session with interactive discussion and evaluation of the DBS lead placements. A second ~2 h session was focused on the sEEG lead placements. The HoloSNS plans were typically finalized ~1 week prior to the surgery date. The electrode trajectories of the HoloSNS plans were then saved in the stereotactic coordinate system of the commercial surgical planning workstation that would be used for the procedure.

The first 6 subjects in NCT03437928 were implanted using the ROSA ONE Brain system (Zimmer Biomet). To transfer the plan defined in HoloSNS into the ROSA system, the electrode trajectories were “burned into” (i.e. black voxels along each trajectory) a copy of the surgical targeting T1w MRI. This HoloSNS electrode trajectory mask image was then loaded into the ROSA planning software to verify the accuracy and vascular safety of the HoloSNS plan on an FDA-approved planning station. The final surgical plan was then defined within the ROSA system by the surgeon of record for the case. The final lead trajectories defined in ROSA were typically indistinguishable from those designed in HoloSNS. However, the FDA-approved ROSA system represented our “gold standard” from a safety perspective, so final decisions on the exact trajectory angles for blood vessel avoidance were always made within that system.

## 3. Results

In late 2019 and early 2020, we (Sheth, Pouratian, Bijanki, McIntyre, Noecker, and Mlakar) convened in-person to perform the first prospective patient-specific group-based holographic neurosurgical plans based on connectomic hypotheses [14] (Fig. 1A). During those initial meetings, we defined 14 intracranial electrode trajectories (4 DBS and 10 sEEG) within the pre-operative imaging data for the first subject in clinical trial NCT03437928. HoloSNS facilitated a unique 3D appreciation of the brain anatomy, as well as the mutual connectivity (i. e. simulated axonal streamlines) between the DBS targets (SCC and VC) and the sEEG recording contacts, which we distributed throughout prefrontal cortex [17] (Fig. 2). This allowed us to interactively explore how different sEEG electrode trajectories would facilitate the post-operative DBS testing of different connectomic hypotheses. Given the novel anatomical and surgical insights that were derived from this initial clinical test of HoloSNS, we elected to continue its use for the 5 additional subjects that have been implanted in NCT03437928 to date. Each subject followed the same general surgical planning workflow described below.

The 4 DBS lead trajectories in each subject were defined using a strategy to engage a precise target volume within the SCC and VC that had the maximal joint probabilities of engaging white matter tracts related to each target [8,14] (Fig. 2). An important entry point consideration for the VC DBS electrode trajectories was the desire to traverse “down the barrel” of the anterior limb of the internal capsule, avoiding the caudate medially and the putamen laterally. For the SCC DBS electrode trajectories, we avoided the frontal horn of the lateral ventricle. For both DBS targets, we carefully studied the vascular pattern using both 3D blood vessel models and 2D slice-by-slice scrutiny of the T1w MRI with contrast to create trajectories avoiding vascular collision.

Planning the 10 sEEG trajectories in each subject was a bit more abstract and focused on four general considerations (Fig. 2). First, the desire to sample key prefronto-temporal regions (e.g., dorsolateral prefrontal cortex (dlPFC), dorsal cingulate cortex (dACC), orbitofrontal cortex (OFC), ventromedial and ventrolateral prefrontal cortex (vmPFC, vlPFC), amygdala, etc.). Second, maximize grey matter sampling of the brain regions connected to the DBS targets based on connectomic mapping, while minimizing the total number of trajectories. Third, spatially distribute trajectories to avoid under- or over-sampling of the brain regions. Fourth, plan feasible entry points with considerations of: 1) cosmesis (avoid incisions in front of the hair line), 2) anchor stability (avoid entry points with thin bone and thick soft tissue with trajectories as perpendicular to the skull as possible), 3) infection risk (avoid entry points near the DBS leads and eventual subcutaneous path of the DBS extension wires that could contaminate those permanent implants), and 4) safety (avoid vascular collision and enter on a gyrus).

The ability to visualize the simulated white matter connections, as well as the skull/scalp/vessel models in 3D, in combination with the ability to collaboratively interact as a research team, was critical for the sEEG planning (Fig. 2). We chose entry and target points for the sEEG leads to sample cortical regions with predicted connections to the DBS leads. To do so, we simulated activation volumes on each contact of the DBS leads to visualize the streamlines connecting the stimulation site and cortex. Because sEEG entry points are in the lateral cortical surface, we narrowed down the vast dlPFC and vlPFC regions to those with strong predicted connectivity to the DBS leads and, in the more recent participants, the area of dlPFC with inverse functional connectivity with the SCC (i.e. anti-correlated rs-fMRI activity). Similarly, we chose target points in dACC, vmPFC, OFC, and amygdala with predicted connectivity to the DBS leads. Holographic visualization greatly facilitated this facet of the surgical planning.

The sEEG electrode entry points on the head, relative to the DBS leads, were also an important consideration for these patients (Fig. 2). The sEEG electrodes are placed percutaneously with anchors. The DBS leads are placed via curvilinear incisions through 14 mm burr holes to accommodate a burr hole cover to anchor the lead and maintain its rotational orientation. Therefore, we used the HoloSNS skull and skin models to visualize the positions of the DBS and sEEG leads and approximate the shape and location for the DBS incisions. We also used the skin model to plan the course of the subcutaneous DBS lead extensions that are placed and externalized during the initial surgery [14]. These externalization sites (10 sEEG electrodes and 4 DBS lead extensions) are considered contaminated, so we wanted to ensure that the permanent hardware and their incisions were a safe distance away from the sEEG anchors. An additional cable routing consideration that we visualized was the location of the eventual bilateral retro-auricular incisions that would be placed during the second surgery and used to route the permanent internalized DBS lead extensions to the implanted generators following the in-patient monitoring period.

Soon after the first subject in NCT03437928 was implanted, the COVID-19 pandemic changed the world. That new reality made group-based in-person surgical planning impractical during 2020 and 2021. Therefore, we adapted HoloSNS to enable remote interaction with the patient-specific holographic brain model (Fig. 1C, D, E, F). We have subsequently found that this “metaverse-based” HoloSNS surgical planning is faster, easier, and more convenient (i.e. no travel, excellent group-based interactivity, and easier to schedule team meetings). As such, all of the following subjects in NCT03437928 were planned remotely (each with 14 total leads). These remote planning sessions typically consisted of 3–4 different remote site locations, all simultaneously interacting within the same holographic session. For example, a planning session from a recent subject consisted of Sheth at his home in Houston, Bijanki at her home in Houston, Pouratian in his office in Dallas, as well as Noecker and McIntyre in his office in Durham (Fig. 1C, D, E, F) (Supplemental Videos 1, 2, 3).

## 4. Discussion

A common theme emerging from invasive neuromodulation technology development efforts is that the diseases we hope to treat with brain stimulation are likely rooted in network dysfunction [23]. As such, scientific research teams are continuously developing new methods and models to characterize these networks on a patient-specific level [11]. However, commercial neurosurgical planning workstations are not necessarily conducive to the integration of novel scientific datasets into their proprietary systems. In turn, there is a need for alternative research analysis tools that can model the 3D anatomy of the patient with the latest scientific imaging and modeling results to help guide neuromodulation therapy development efforts. HoloSNS was designed to address those research needs, and provide an unparalleled group-based holographic visualization environment for clinical teams to plan their stimulation experiments [17]. The purpose of this report was to describe our first experiences with the prospective use of HoloSNS in support of a novel clinical trial (NCT03437928).

In attempts to better define the underlying neural targets for therapeutic DBS, investigators are rapidly expanding their use of imaging-based and electrophysiology-based approaches to map brain networks of interest for the treatment of psychiatric disorders [e.g. [13,14]]. In parallel, the development of patient-specific connectomic modeling techniques, integrated with neural stimulation models, are facilitating a better understanding of how different parts of the brain are modulated by DBS [e.g. [10,24]]. These technical innovations have evolved into the prospective application of patient-specific connectomic DBS surgical targeting strategies [18], which have demonstrated impressive improvements in the outcomes of exploratory clinical trials [9].

Early examples of connectomic DBS surgical planning successfully relied on traditional computer screen based visualization [18]. However, as connectomic DBS hypotheses have become more elaborate, and the number of implanted electrodes has increased, we identified a need for new techniques to integrate and visualize the wide variety of 3D brain imaging results and computational modeling datasets that are becoming relevant to modern DBS surgical planning [17]. Along this line, holographic visualization represents a logical evolution for neurosurgical planning systems and wide-ranging commercial efforts are actively developing new tools for 3D visualization. Nonetheless, commercial planning systems are typically designed to be run by a single neurosurgeon user, which hinders opportunities for team-based collaborative interaction when creating complex multi-nodal multi-electrode surgical plans. Further, the integration of novel research-based imaging and modeling results into their closed software environments can be challenging. Therefore, research tools like HoloSNS provide an augmentative option for use in complex cases with brain neuromodulation devices.

One important research objective in clinical trial NCT03437928 is to use structural connectivity models to assist in the electrode placement planning and stimulation parameter selection processes. However, there are many different methods for representing brain connectivity within an individual subject and it is currently unclear which method(s) provide the most clinically meaningful information. For example, the Mayberg group has found great success with patient-specific tractography for SCC DBS surgical planning [9], while others advocate for the use of normalized connectomes for VC DBS analyses [25]. In turn, we found that a valuable aspect of our HoloSNS analyses was the opportunity to simultaneously visualize different scientific models of connectivity within each patient-specific model, evaluate their congruence (or non-congruence), and simulate how DBS at different contacts might alter the directly activated circuitry (Fig. 2). These model-based predictions can then later be compared with the sEEG evoked potential recordings to help verify (or refute) their connectomic accuracy [16]. As such, the burgeoning field of connectomic DBS surgical planning is only beginning to define the modeling standards that will be necessary to establish wider-scale clinical relevance for the approach. Therefore, we propose that research tools like HoloSNS provide an important clinical testing ground for the comparison and evaluation of different connectomic modeling methods.

While HoloSNS is an interesting technological concept, and we have found it to be scientifically useful, the tool is not without its limitations. First, HoloSNS is an academic research prototype without any form of regulatory approval. All of our work was performed under IRB approval, and we have undertaken extensive validation testing of the coordinate systems and data co-registration processes. In addition, the explicit accuracy of the data in HoloSNS is equivalent when seen as a hologram or on a computer screen. Nonetheless, the data co-registration issues that govern any brain imaging visualization tool are a key limitation to this kind of work [18]. One could also consider the specific assortment of datasets and modeling methods we employed in this study as a limitation of the work. For example, there are many different anatomical atlases [26], connectomic datasets [27], and DBS modeling methods [28] available in the literature. Nonetheless, HoloSNS is designed to be agnostic to the datasets that are loaded into the system and it is capable of accommodating new modeling methods as they become available. Another limitation is the visualization constraints of the currently available HMDs, which have a finite resolution and limited field of view. However, we have found that users naturally adapt to those constraints after a few minutes of experience with HMDs.

Other factors to consider are the real time and effort costs associated with developing detailed research-based patient-specific brain models and then facilitating their holographic visualization. Justification for those costs must be rooted in the specific research goals of the clinical study. This is because it is not yet clear if any therapeutic outcome value can be attributed to these advanced modeling and visualization exercises. Along this line, the broader applicability of using HoloSNS patient-specific models on a large scale is not currently possible due to the many steps associated with their construction and the unique expertise required for their implementation. Nonetheless, we consider the primary use case for HoloSNS to be in the initial clinical evaluation of next generation neurosurgical planning concepts. If those experiences are then deemed successful, they can help guide the future development of software tools that are intended to service a much larger user base.

It should also be noted that universally-defined metrics or algorithms for defining an “optimal” electrode trajectory do not exist (aside from general safety considerations). Possibly one day, connectomic modeling methods could provide a validated basis for optimizing patient-specific electrode placements. However, at present the clinical positioning of DBS and/or sEEG leads largely remains a surgical art, albeit one guided by science and experience. Therefore, the basic points of using HoloSNS in NCT03437928 were to provide explicit co-registration of the latest connectomic datasets into the clinical planning process (Fig. 2), and expand opportunities for the research team to interactively discuss the surgical plan within the context of the scientific data (Fig. 1).

## 5. Conclusion

This report describes our first experiences with the prospective use of a group-based holographic stereotactic neurosurgery research tool (HoloSNS) in support of the placement planning for DBS and sEEG leads in an exploratory clinical trial focused on therapy development for TRD. HoloSNS is a visualization tool for clinical research teams to analyze their experiments and integrate novel connectomic datasets into the patient-specific surgical planning process. While the concepts of connectomic DBS surgical planning have already found utility within the context of traditional computer screen planning environments, we found that the 3D nature of the surgical procedure, brain imaging data, and connectomic modeling results, all highlighted the value of holographic visualization in support of these unique clinical experiments.

## Supplementary Material

1

2

3
